# Pulsed Laser Phosphorus Doping and Nanocomposite Catalysts Deposition in Forming a-MoS*_x_*/NP-Mo//n^+^p-Si Photocathodes for Efficient Solar Hydrogen Production

**DOI:** 10.3390/nano12122080

**Published:** 2022-06-16

**Authors:** Vyacheslav Fominski, Maxim Demin, Dmitry Fominski, Roman Romanov, Oxana Rubinkovskaya, Petr Shvets, Aleksandr Goikhman

**Affiliations:** 1 National Research Nuclear University MEPhI (Moscow Engineering Physics Institute), Kashirskoe sh. 31, 115409 Moscow, Russia; dmitryfominski@gmail.com (D.F.); limpo2003@mail.ru (R.R.); oxygenofunt@gmail.com (O.R.); 2 Immanuel Kant Baltic Federal University, A. Nevskogo St. 14, 236016 Kaliningrad, Russia; sterlad@mail.ru (M.D.); pshvets@kantiana.ru (P.S.); aygoikhman@gmail.com (A.G.)

**Keywords:** amorphous molybdenum sulfide, Si-based photocathode, laser-based technique, n^+^p-junction, hydrogen evolution reaction

## Abstract

Pulsed laser deposition of nanostructured molybdenum sulfide films creates specific nonequilibrium growth conditions, which improve the electrocatalytic properties of the films in a hydrogen evolution reaction (HER). The enhanced catalytic performance of the amorphous a-MoS*_x_* (2 ≤ *x* ≤ 3) matrix is due to the synergistic effect of the Mo nanoparticles (Mo-NP) formed during the laser ablation of a MoS_2_ target. This work looks at the possibility of employing a-MoS*_x_*/NP-Mo films (4 and 20 nm thickness) to produce hydrogen by photo-stimulated HER using a p-Si cathode. A simple technique of pulsed laser p-Si doping with phosphorus was used to form an n^+^p-junction. Investigations of the energy band arrangement at the interface between a-MoS*_x_*/NP-Mo and n^+^-Si showed that the photo-HER on an a-MoS*_x_*/NP-Mo//n^+^p-Si photocathode with a 20 nm thick catalytic film proceeded according to a Z-scheme. The thickness of interfacial SiO*_y_*(P) nanolayer varied little in photo-HER without interfering with the effective electric current across the interface. The a-MoS*_x_*/NP-Mo//n^+^p-Si photocathode showed good long-term durability; its onset potential was 390 mV and photocurrent density was at 0 V was 28.7 mA/cm^2^. The a-MoS*_x_*/NP-Mo//n^+^p-Si photocathodes and their laser-based production technique offer a promising pathway toward sustainable solar hydrogen production.

## 1. Introduction

Because of their promising potential in hydrogen production technology by electrochemical (EC) and photoelectrochemical (PEC) water splitting, amorphous molybdenum sulfides (a-MoS*_x_*), such as nanoclustered Mo_3_S_4_/Mo_3_S_12_/Mo_3_S_13_, have been widely developed and investigated since 2011 [1,2,3,4,5]. These nanomaterials have high concentrations of catalytically active sites, whose nature is still the subject of debate [6,7,8]. In the local packing of Mo and S atoms in an amorphous/clustered a-MoS*_x_* matrix, catalytically active sites, such as terminal disulfide units, are uniformly distributed over the volume and surface of the catalyst. This distribution simplifies the formation of a catalytic layer on the carrier/electrode surface compared to 2D, quasi-2D, and texture nanocrystalline MoS_2_ catalytic layers. Nanocrystalline MoS_2_-based catalysts require a nanostructure in which the basal planes of the laminar crystalline 2H-MoS_2_ structure are perpendicular to the carrier surface [9,10,11]. In addition, only under this condition is it possible to achieve the high surface density of active sites localized at the edge sites of such a laminar packing of atomic planes [12,13]. It is difficult today to give preference to any modification of molybdenum sulfides (a-MoS*_x_*_≥2_, 2H-MoS_2_, 1T-MoS_2_) when forming high-quality catalysts for (photo)electrochemical water splitting to produce hydrogen. Electrode characteristics depend on a large number of factors: the structural and chemical state of the catalytic layers (especially when forming hybrid/composite nanomaterials), the nature of the support/electrode, and the state of the interface between the catalyst and the support/electrode [14,15,16,17].

Amorphous molybdenum sulfides (a-MoS*_x_*) have been identified as active non-noble-metal HER electrocatalysts. Most often, a-MoS*_x_* films are synthesized by chemical, thermal, and (photo)electrochemical techniques [18,19,20,21,22,23]. During these processes, the local packing of Mo and S atoms forms under the influence of a chemical driving force. The chemical composition and concentration of the precursors significantly affect the chemical composition of the films (the *x* = S/Mo ratio). Sulfur concentration is generally assumed to influence the catalytic properties and increase the sulfur content (2 ≤ *x* ≤ 6) to improve catalytic activity. However, sulfur content gradually depletes over time, leading to a slow deactivation of the a-MoS*_x_* film [24,25].

Physical vapor deposition techniques of a-MoS*_x_* film formation (PVD: magnetron and pulsed laser deposition) seem to be more environmentally friendly and flexible than chemical techniques. Of particular interest here is pulsed laser deposition (PLD) [26,27,28,29,30]. Only MoS_2_ powder is required to obtain a-MoS*_x_* films; the powder is pressed into the target wafers under normal conditions. This technique allows flexible adjustment of the structure, morphology, and chemical composition of the deposited a-MoS*_x_* films [27,31,32,33]. The MoS_2_ target is irradiated by high-intensity laser pulses of nanosecond duration. Then, the plasma–vapor flow formed during laser ablation is deposited under vacuum conditions on a substrate mounted at a distance from the target. The nonequilibrium nature of pulsed laser deposition leads to the synthesis of self-supported hierarchical nanoarchitectures. Fominski et al. [34,35,36] and Giuffredi et al. [33] have investigated the physics behind the PLD mechanism, making it possible to identify several processes exclusively characteristic of PLD. These are ion bombardment, gas-phase nucleation, attachment of defective molybdenum sulfide clusters, and Mo nanoparticle (NP-Mo) and oxysulfide nanophase formation. The degree to which these processes influence the properties of the formed a-MoS*_x_* catalysts and the state of the interface with the support (electrode) largely depends on the modes of the pulsed laser ablation of the MoS_2_ target, as well as the laser plume propagation and deposition conditions.

Numerous studies have shown that an a-MoS*_x_* thin-film catalyst can be successfully applied to create an efficient Si-based photocathode since this catalyst can solve the problem of slow HER kinetics on silicon surfaces [1,3,9,37,38,39,40]. Silicon is an ideal photocathode semiconductor material with a suitable optical band gap (1.1 eV), and it can absorb light with a wavelength of less than 1100 nm in the solar spectrum. Most of the studies used (photo)electrochemical synthesis techniques to deposit a-MoS*_x_* films on Si substrates. When forming the films, requirements for the chemical composition of the films and the state of the Si surface were considered. It was also essential to preclude SiO_2_ formation. Such photocathodes are commonly formed on p-Si as the configuration of energy bands in this type of silicon causes electrons formed under illumination to migrate to the silicon surface and holes from the surface [38,39]. Although nanocrystalline MoS_2_ catalysts usually have n-type conductivity [41,42], relatively little research has been carried out on the conductivity type and energy band structure for a-MoS*_x_* films formed by different techniques. Zhang et al. [37] have found that, in an a-MoS*_x_* catalyst obtained by electrodeposition, the Fermi level is located near the conduction band. So, the mechanism of charge transfer under illumination provides efficient electron migration through the p-Si interface into the volume of a-MoS*_x_* and onto the catalyst surface. The performance of an a-MoS*_x_*//Si photocathode can be improved by taking advantage of the photovoltaic effect in the formation of an np- or n^+^p-junction in the near-surface layer of p-Si [3,43,44]. Yet, when using np-Si and n^+^p-Si electrodes, it is critical to consider the possible configuration of energy zones during the contact formation with a-MoS*_x_*.

Fominski et al. [32] have defined optimal conditions for obtaining MoS*_x_*/NP-Mo by PLD: these films have a high catalytic HER activity in an acidic solution. It has also been established that Mo nanoparticles exert a positive synergetic effect on the catalytic activity of S_2_-sites in an a-MoS_3_ matrix. Moreover, the possibility of using these catalytic films to activate PEC processes on photocathodes made of tungsten trioxide thin films is entertained in [34]. Despite this, the characteristics of the band structure of a-MoS*_x_*/NP-Mo catalytic films formed by PLD and their possible applications in creating Si-based photocathodes for hydrogen production remain unclear. In addition, this study aims to obtain a-MoS*_x_*/NP-Mo//n^+^p-Si photoelectrodes using laser-based techniques and investigate their efficiency and stability in PEC hydrogen production in an acid solution. Relatively thin (4 nm) and thicker films (20 nm) of the a-MoS*_x_*/NP-Mo catalyst were created, for which different mechanisms were possible to transfer electrons (formed under illumination) from the silicon to the catalyst surface. Laser-based techniques help apply a-MoS*_x_*/NP-Mo catalytic films and modify the p-Si wafer surface layer by doping it with phosphorus atoms [45,46]. The simplest doping technique was chosen, namely the pulsed laser irradiation of a p-Si wafer in an H_3_PO_4_ acid solution. Previous research has not addressed the efficiency of the n^+^p junction formation or its applicability in obtaining a-MoS*_x_*/NP-Mo//n^+^p-Si photoelectrodes. This paper presents the results of modifying the surface layer of a p-Si wafer, which could influence the performance of a-MoS*_x_*/NP-Mo//n^+^p-Si photocathode.

## 2. Materials and Methods

### 2.1. Pulsed Laser Doping of p-Si Wafers with Phosphorous

Single-crystal B-doped (p-type, (100) oriented) 100 mm diameter silicon wafers (300 mm thick, 10 Ω cm resistivity) were cut into 1 × 1 cm^2^ pieces and sonicated in ethanol and distilled water for 15 min each to remove any contaminants. The samples were then dipped in buffered HF to remove the native oxide. The surface of the p-Si wafer was doped with phosphorus by pulsed laser irradiation of the wafer in an orthophosphoric acid solution (H_3_PO_4_) on a VENO FIBER VPG unit (VENO, Sant-Petersburg, Russia). A fiber laser with a wavelength of 1064 nm and a pulse duration of ~100 ns was used. The diameter of the laser beam was 60 μm. After focusing radiation, the laser fluence in the exposure zone was ~12 J/cm^2^; the pulse repetition rate was 70 kHz. The substrate scanning speed during irradiation was set to 500 mm/s, providing a partial overlap of the pulsed laser exposure zones. A sample area of 0.7 cm^2^ was irradiated ten times. The p-Si wafer was placed at the bottom of a glass flask filled with a 40% H_3_PO_4_ solution and laser-irradiated through the solution. A mode was chosen in which the resulting gas bubbles had no appreciable effect on the laser exposure of the sample. Microscopic analysis of the silicon surface morphology after laser treatment pointed to the melting of the Si surface under pulsed laser exposure. After laser treatment in an acid solution, the samples were washed in distilled water and then used to produce photocathodes.

### 2.2. Preparation of a-MoS_x_/NP-Mo//n^+^p-Si Photocathodes

To obtain a-MoS*_x_*/NP-Mo//n^+^p-Si photocathodes, n^+^p-Si samples were dipped in buffered HF to remove the native oxide and hydrogen terminate the surface. The samples were then sonicated in ethanol and distilled water to remove any contaminants. The back of the n^+^p-Si samples was covered by a thin Au layer to obtain an Ohmic contact with p-Si. A 30 nm-thick Au film was prepared by PLD from the Au target at room temperature of the samples. Laser ablation of the target was performed using second harmonic emission from a Solar LQ529 laser (Solar LS, Minsk, Belarus) in buffer gas (Ar) at a pressure of 2 Pa. The formation of the rear contact to the p-Si sample took 1–2 h (all the manipulations taken into account); this could cause oxidation of the n^+^-Si side surface. After applying the Au film, the samples were again chemically treated and cleaned in a hydrofluoric acid solution and then distilled water.

Pulsed laser deposition of a-MoS*_x_*/NP-Mo films was carried out according to the standard PLD configuration used earlier for high active a-MoS*_x_*/NP-Mo electrocatalytic film preparation [32]. Laser radiation was directed at an angle of 45° to the target surface. The n^+^p-Si sample for film deposition was installed normal to the expansion of the laser ablation plume and parallel to the target surface. The substrate was 4 cm away from the target. A Solar LQ529 laser (Belarus) was used for ablating the targets made of pure (99.9%) pressed MoS_2_ powder. Laser radiation had a wavelength of 1064 nm, a pulse duration of 15 ns, a pulse repetition rate of 20 Hz, a pulse energy of ~50 mJ, and a laser fluence of ~7 J/cm^2^. The PLD vacuum chamber was pumped out by a turbomolecular pump to a pressure of ~10^−3^ Pa. Buffer gas (Ar) was then let into the chamber to a pressure of 11 Pa, and the MoS_2_ target was ablated by laser. Buffer gas had a considerable influence on the S concentration in the films. The pressure was chosen so as to obtain a composition in which *x ~*3. The deposition times of a-MoS*_x_*/NP-Mo films on the n^+^ side of n^+^p-Si sample were 2 and 10 min. These intervals resulted in the formation of films with an average thickness of 4 and 20 nm, respectively.

A conductor was pressed onto the Au-coated surface of the obtained a-MoS_x_/NP-Mo//n^+^p-Si samples. This side and the edges of the silicon wafer were then sealed with a layer of epoxy resin; this created an active surface area of 0.7 cm^2^.

### 2.3. Structural, Chemical, and Energy Band Alignment Studies

The surface morphology of laser P doped p-Si wafers before and after the a-MoS*_x_*/NP-Mo film deposition was studied by scanning electron microscopy (SEM, Tescan LYRA 3, Brno, Czech Republic). The silicon wafers were split by mechanical impact, and a cross-section SEM study of the surface layer was carried out. Rutherford back-scattering spectroscopy (RBS) and elastic recoil detection analysis (ERDA) techniques were employed to determine the influence of laser treatment on the monocrystalline structure of a silicon wafer and the possible concentration of hydrogen (H) in its surface layer. The energy of helium ions in the analyzing beam was 1.5 MeV, and the detector resolution was 20 keV. The RBS spectra were recorded in the random and aligned (along the <100> crystallographic axis) configurations. The ERDA spectra were recorded in the following configuration: α = 80°, β = 80°, θ = 20°. The measured spectra data were processed using the Simnra software (Max-Planck-Institut für Plasmaphysok, Garching bei München, Germany).

The laser doping effect was assessed based on measuring the electric properties of the laser-treated silicon samples. The p-Si and n^+^p-Si wafers with a Au back contact were placed onto a conductive ground electrode. A W probe of a diameter of ~10 µm was linked to the surface of the Si wafer. The potential of the probe was between –1.5 and 1.5 V. The analyzed area was controlled by an optical microscope. The *I-U* dependence was measured under dark conditions and when the sample was irradiated by the lamp of the optical microscope.

To reveal their structural features, 4 nm thick a-MoS*_x_/*NP-Mo films were separated from the Si substrate in an alkaline solution. Then, they were transferred onto metal grids and studied by transmission electron microscopy and selected area electron diffraction (TEM and SAED, JEM-2100, JEOL, Tokyo, Japan). The structure and composition of 20 nm thick a-MoS*_x_/*NP-Mo film deposited on n^+^p-Si were studied by cross-section STEM/SAED, high-resolution TEM (HRTEM), and annular dark-field imaging (HAADF) performed in parallel with energy-dispersive X-ray spectroscopy (EDS) acquisition, using a Carl Zeiss Libra 200FE microscope. The chemical states of the surface of a-MoS*_x_/*NP-Mo//n^+^p-Si samples were studied by X-ray photoelectron spectroscopy (XPS). XPS spectra were obtained by a Theta Probe Thermo Fisher Scientific spectrometer with a monochromatic Al Kα X-ray source (hv 1486.7 eV) and an X-ray spot size of 400 μm. The spectrometer energy scale was calibrated using Au 4f_7/2_ core level lines located at a binding energy of 84.0 eV. TEM and XPS studies were carried out after the a-MoS*_x_/*NP-Mo film deposition and after their testing in PEC HER.

XPS measurements were performed to construct band alignment in the semiconductor a-MoS*_x_*/NP-Mo//n^+^p-Si heterostructures. Determining the shift between the core levels of the semiconductors in the heterojunction made it possible to calculate the valence band offset (*VBO*). For estimating the conduction band offset (CBO), the published and/or previously obtained energy band gap data were used. To calculate the *VBO* in the heterostructure, a series of measurements were performed. At first, the XPS spectra of Mo3d for a 20 nm thick a-MoS*_x_*/NP-Mo film and Si2p core levels were measured along with the spectra of the valence bands. The spectra of the Mo3d and Si2p core levels were then measured for the a-MoS*_x_*/NP-Mo//n^+^p-Si heterostructure, in which the thickness of the upper layer (a-MoS*_x_*) was ≤ 4 nm. After that, the *VBO* value for heterojunctions was calculated as:

*VBO* = (*E*_*Mo3d5*/*2*_ − *VBM_Mo_*)_bulk_ − (*E*_*Si2p3*/*2*_ − *VBM_Si_*)_bulk_ − (*E*_*Mo3d5*/*2*_ − *E_Si2p3/2_*)*_interface_*,

where *VBM_Mo_* and *VBM_Si_* are the energies of the upper edge of the valence band for a-MoS*_x_* and Si, respectively. “Interface” stands for the spectra of heterojunctions, and “bulk” for the spectra of thicker films and the Si substrate.

### 2.4. Photoelectrochemical Measurements of HER

To investigate the PEC characteristics of a-MoS*_x_*/NP-Mo//n^+^p-Si samples in HER, the samples were illuminated by radiation from Xe lamps with a power of 100 W in a 0.5 M H_2_SO_4_ aqueous solution. The light intensity was maintained at 100 mW/cm^2^. A three-electrode configuration was used to measure the photo-activated current in an electric circuit with modified cathodes. The polarization curves were measured using linear sweep voltammetry (LSV) with the applied potential changing from −200 to +400 mV and a scan rate of 2 mV/s. When measuring the LSV curves and the time evolution of the photocurrent, the light source was turned on and off. For chronoamperometry measurements, the potential of the tested samples was maintained at zero level (relative to the reversible hydrogen electrode, RHE). Electrochemical impedance spectroscopy (EIS) was performed at a potential of 0 V (RHE) in the frequency range of 10^5^ to 0.1 Hz, with a perturbation voltage amplitude of 20 mV. The PEC HER characteristics of n^+^p-Si (without a catalytic film) and Pt//n^+^p-Si electrodes were measured for comparison.

## 3. Results

### 3.1. The Surface Morphology and Structure of Laser-Doped n^+^p-Si

**Figure 1:**
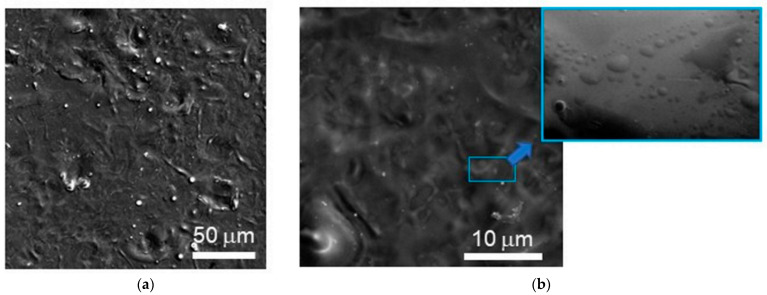
SEM images of the surface of a p-Si wafer after pulsed laser P-doping in an H_3_PO_4_ solution measured with (**a**) low and (**b**) higher magnification. The insert in (**b**) shows a periodic surface structure consisting of rounded nano-hills.

**Figure 2:**
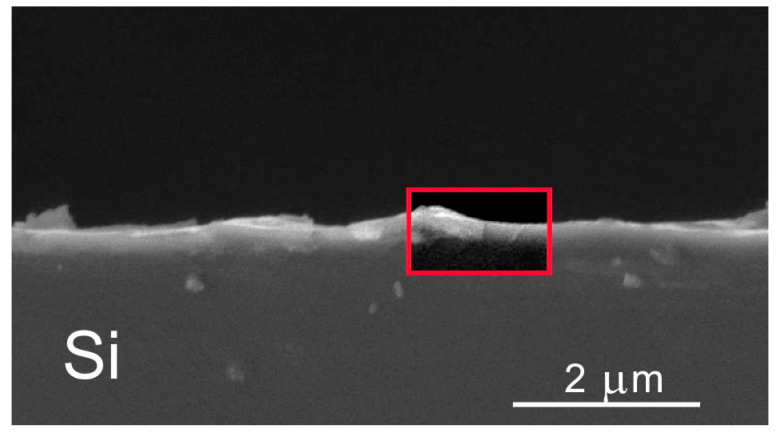
Cross-section SEM image of a pulsed laser P-doped p-Si wafer.

Figure 1a,b shows the results of the SEM investigation after laser treatment in orthophosphoric acid. Laser exposure caused the surface layer of the wafer to melt. After the melt solidified, folds (“frozen” waves) were formed, their size reaching several micrometers across the surface. A SEM study of the cross-section fracture of the wafer showed that the height of the waves did not exceed 500 nm. Under higher magnification, one can see that rounded irregularities (hills) are formed on the surface of the wavelike folds whose lateral size varies from 100 to 200 nm (insert in Figure 1b). There is a certain periodicity in the arrangement of such irregularities on the surface of the sample. The formation of periodic structures of this type may be due to the instability of the interface between the surface of the molten wafer and the acid solution when exposed to the laser pulse [47]. SEM analysis of the cross-section of this sample showed that the fracture did not have any morphological features at depths greater than 500 nm (Figure 2). Only the near-surface layer less than 500 nm thick showed signs of brittle fracture. This indicates that the principal laser-induced structural disturbances in the Si monocrystal were localized in the surface layer up to 500 nm thick.

**Figure 3:**
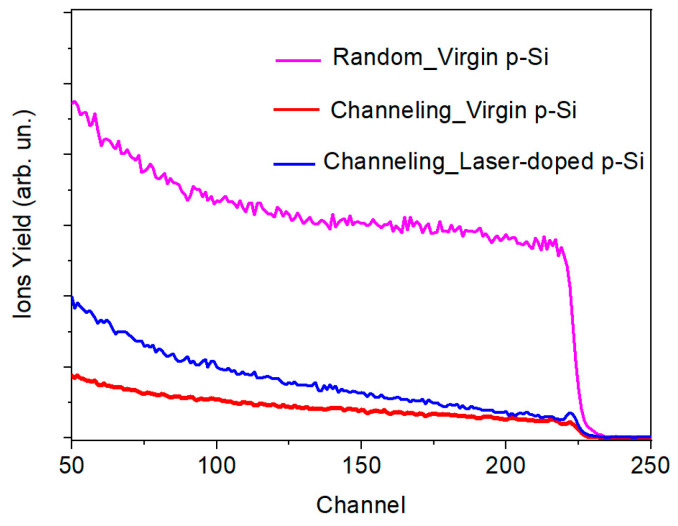
Random and aligned (channeling along <100>) RBS spectra measured for p-Si crystal before and after pulsed laser P doping. For virgin and laser-doped Si, random spectra coincided.

Structural investigations of the Si wafer showed that the pulsed laser impact caused slight changes in the monocrystalline structure (Figure 3). The aligned spectra for the irradiated and the virgin crystals differed slightly. This pointed to the recovery of the Si crystal lattice after the melting caused by pulsed laser irradiation was of sufficiently high quality. The only difference was that the aligned spectrum of the laser-irradiated silicon peaked around channel 225. This peak is usually explained by the disturbance of the ideal monocrystalline lattice on the silicon surface due to partial oxidation. Mathematical processing of the spectrum showed that the thickness of the disturbed (amorphous/nanocrystalline) surface layer was ~4 nm. In the virgin crystal, which contained a layer of natural silicon oxide, no such noticeable disturbance of crystallinity in the surface layer occurred. This indicated that the laser treatment caused more significant surface modification than oxidation in air. Thus, it was necessary to investigate the possibility of hydrogen introduction during laser treatment in an acid solution and its effect on the surface layer structure.

ERDA studies showed that the Si surface of the sample subjected to laser irradiation in H_3_PO_4_ contained ~2 × 10^15^ cm^−2^ H atoms (Figure S1). After treatment in an HF solution, the hydrogen content decreased slightly. Additional investigation of a pure Si wafer with natural oxide revealed that about the same amount of hydrogen could be present on its surface. The presence of hydrogen might be due to the adsorption of hydrocarbon molecules or water on the surface of the studied samples. Therefore, the formation of a thin layer of hydrogenated silicon suboxide during the laser doping of silicon in orthophosphoric acid seems unlikely.

**Figure 4:**
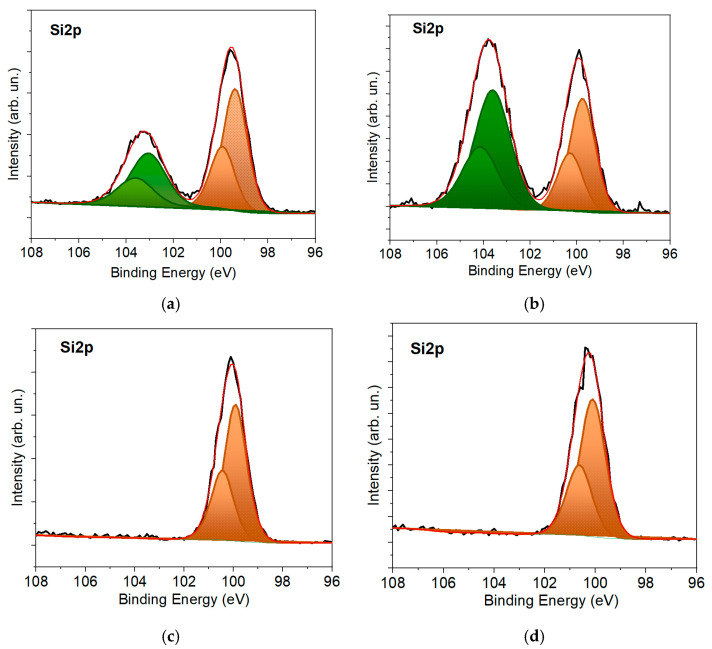
XPS Si2p spectra for (**a**) virgin p-Si with native oxide layer; (**b**) laser-doped n^+^p-Si with formed oxide layer; (**c**) virgin p-Si after HF solution treatment, and (**d**) laser-doped n^+^p-Si after HF solution treatment.

Figure 4 shows sections of XPS spectra for a silicon wafer after laser treatment in an H_3_PO_4_ solution. The spectra were measured after the laser treatment of silicon and the etching of the irradiated sample in HF solution. The etching in HF was performed immediately before placing the sample in the XPS spectrometer. For comparison, the spectra are shown for the unirradiated part of the Si wafer. It can be seen that pulsed laser treatment was accompanied by silicon oxidation more noticeably than natural oxidation in the air (Figure 4a). This was manifested by an increase in the relative intensity of the peak corresponding to the Si-O states at a binding energy of ~103.5 eV. This result is in accord with the RBS analysis of the Si wafer. The laser treatment of the silicon also led to a shift of peaks corresponding to Si-Si and Si-O states to the region of a higher binding energy. Treating unirradiated and laser-doped silicon in an HF solution caused the removal of the silicon oxide layer. The shift between the peaks corresponding to Si-Si bonds in these samples was generally preserved after etching. The S2p_3/2_ peak shifted from 99.63 eV to 99.81 eV, which could be due to a change in the conductivity type of silicon following P doping. The position of the Si2p peak in n- and p-type Si can differ by ~0.18 eV [48,49]. The preservation of such a shift after etching in the HF solution indicated that, unlike oxidation, doping occurred in the deep layers of silicon.

The methods applied in chemically analyzing the silicon sample’s surface did not reveal the penetration of P atoms into the silicon substrate after laser treatment: phosphorus concentration did not exceed 0.1 at.%. Additional studies of the electrical properties of the silicon substrates before and after pulsed laser doping showed that the initial p-Si demonstrated high resistance to the current flow in both the forward and reverse directions of the current (Figure S2). This resistance could be due to both the energy barrier between p-Si and W and the resistance of the natural silicon oxide layer. The character of the *I*(*U*) dependencies did not change when illuminated. Pulsed laser doping of p-Si caused a significant change in electrical properties. This change manifested itself in a noticeable decrease in resistance to the current flow at negative voltages, as well as in the current dependence on illumination at positive potential. The character of *I(U)* dependencies of laser-doped p-Si pointed to the formation of an n^+^p junction in the p-Si as a result of phosphorus penetration during pulsed laser treatment in an H_3_PO_4_ solution. The type of *I(U)* dependencies could be influenced by the energy barrier characteristic of a W contact with p-Si. The effect of this barrier, however, can be leveled if Si is doped heavily enough (6 × 10^19^ cm^−3^) to make electron tunneling possible. The thickness of the layer through which tunneling is possible does not exceed 2.5−5 nm. The shift of the curve under the lamplight of the optical microscope and the increase in the current at the reverse bias indicate that the n^+^p-junction acts as a power generator.

### 3.2. The Composition and Structure of MoS_x_/NP-Mo Films on n^+^p-Si

#### 3.2.1. The 4 nm Thick Film

**Figure 5:**
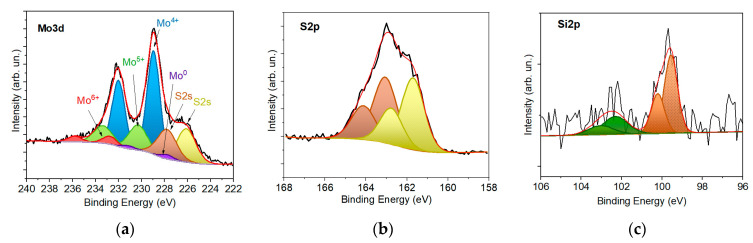
XPS (**a**) Mo3d, (**b**) S2p, and (**c**) Si2p spectra measured for a 4 nm thick a-MoS*_x_*/NP-Mo film prepared by PLD on an n^+^p-Si wafer previously subjected to HF solution treatment.

Figure 5 shows sections of the XPS spectra measured for relatively thin (4 nm thick) MoS*_x_*/NP-Mo films on n^+^p-Si substrates. XPS analysis was performed on a sample previously exposed to air for about a day following PLD. As Mo3d spectrum analyzed, it was assumed that it consisted of doublets corresponding to four different valences: Mo^0^, Mo^4+^, Mo^5+^, and Mo^6+^. Metallic Mo^0^ (a Mo3d_5/2_ peak at 227.8 eV) was caused by the deposition of Mo nanoparticles. The Mo^4+^ state was due to the chemical bonds with the sulfur atoms in a MoS*_x_* compound at *x* ≥ 2 [50,51]. The binding energy for the Mo3d_5/2_ peak was 228.96 eV. The Mo^5+^ state may arise due to the formation of Mo-S-O clusters [52,53]. The appearance of such Mo states is also possible during the chain packing of -Mo-3S-Mo- atoms in the amorphous structure of MoS_3_ [54]. The binding energy for the Mo3d_5/2_ peak in this compound was 230.3 eV. The Mo^6+^ state was realized in molybdenum trioxides; the binding energy for the Mo3d_5/2_ peak in this compound was 232.75 eV [55].

To decompose the S2p spectra, a traditional approach was used that distinguishes high- and low-binding energies [56,57]. The high-binding energy doublet with the S2p_3/2_ line position at 162.77 eV is associated with the apical S^2−^ and bridging (S_2_^2−^)_br_ ligands in Mo_3_S_13_/Mo_3_S_12_ clusters. In addition, the low-binding energy doublet with the S2p_3/2_ line position at 161.71 eV corresponds to the states of single S^2−^ sulfur atoms in MoS_2_-like clusters and terminal (S_2_^2−^)_tr_ ligands in Mo_3_S_13_/Mo_3_S_12_ clusters.

The XPS spectrum of the sample with a relatively thin a-MoS*_x_*/NP-Mo film showed peaks from the silicon substrate. This was due to the depth of the photoelectron escape from silicon exceeding the thickness of the deposited a-MoS*_x_*/NP-Mo film. In the Si spectrum, in addition to the Si-Si doublet, there was a weak peak, possibly a product of Si-O and/or Si-S bonds. A sufficiently rigorous decomposition of this peak was complicated by its weak intensity. Therefore, only the states characteristic of S-O bonds were distinguished in the decompositions of the Si2p spectra. The Si2p_3/2_ peak was located at a binding energy of 99.58 eV.

**Figure 6:**
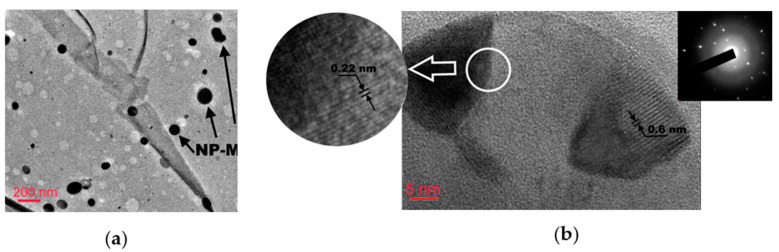
(**a**) TEM image of MoS*_x_*/NP-Mo thin film; (**b**) HRTEM image of Mo nanoparticle. Inserts illustrate crystalline structure of NP-Mo (left) and SAED pattern for the NP-Mo (right), which contains reflections from body-centered cubic lattice of Mo.

When calculating the atomic concentration ratio *x* = S/Mo, only the peaks associated with the Mo^4+^ and Mo^5+^ states and all sulfur states were taken into account. The calculated value was *x*~2.9. XPS analysis revealed the presence of a low content of Mo^0^ in the Mo3d spectra. This could be due to the relatively low surface concentration of Mo nanoparticles in up to 4 nm thick films. Yet the XPS analysis did not reveal the presence of molybdenum metal (Mo^0^) in the Mo3d spectra. This could be due to the relatively low surface concentration of Mo nanoparticles in up to 4 nm thick films. The study of the films by SEM supports this hypothesis. Figure 6a presents planar TEM images of 4 nm thick a-MoS*_x_*/NP-Mo films. TEM images at low magnification show that the films had good mechanical strength and probably covered the substrate as a continuous layer. TEM contrast showed circular areas of dark and light contrast. For the dark areas, SAED patterns and HRTEM images indicated the formation of crystalline Mo particles. The Mo nanoparticles could contain local inclusions of MoS_2_ nanophase possessing a lamellar structure with 0.6 nm interplanar spacing. The formation of relatively light rounded regions of submicron and nanometric size could be due to some Mo nanoparticles not being firmly attached to the thin film surface during laser plume deposition. Such particles could have been removed from the surface during film manipulation. As the thickness of the a-MoS*_x_* matrix increased, the probability of NP-Mo adhesion to the film surface increased since the contact area could expand due to the deformation of a relatively plastic a-MoS*_x_* matrix in the contact area with a Mo particle bombarding the surface of the matrix.

#### 3.2.2. The 20 nm Thick Film

**Figure 7:**
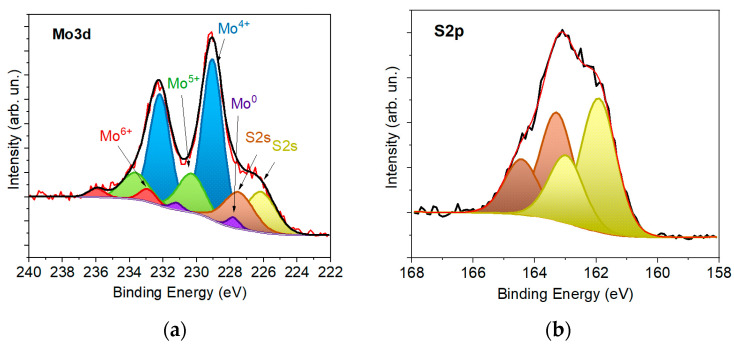
XPS (**a**) Mo3d and (**b**) S2p spectra for a 20 nm thick a-MoS*_x_*/NP-Mo film.

The XPS study of 20 nm thick a-MoS*_x_*/NP-Mo film did not reveal any striking differences in the chemical state of the elements in the films of 4 and 20 nm thickness (Figure 7). According to the XPS measurements, the matrix composition was described by the formula MoS_2.6_. The low intensity of the Mo^0^ peaks suggested that most of the Mo nanoparticles were distributed over the bulk of the a-MoS*_x_* matrix. The Mo nanoparticles located on the film surface were probably covered with a thin MoS*_x_* shell, the thickness of which was comparable to the photoelectron emission depth. Remarkably, for the Mo^4+^ state, the bond energy of the Mo3d_5/2_ peak was 229.05 eV. For a thinner film, this peak was at 228.96 eV. The peak shift could be explained by the incapacity of the Si substrate of relatively thick a-MoS_x_/NP-Mo films to affect the binding energy of electrons in the surface layer of such a film. The difference in the binding energy of the Mo3d_5/2_ peak corresponding to the a-MoS*_x_* compound in a thinner and a thicker film was used later to determine the valence band shift at the a-MoS*_x_*/NP-Mo with n^+^-Si interface.

**Figure 8:**
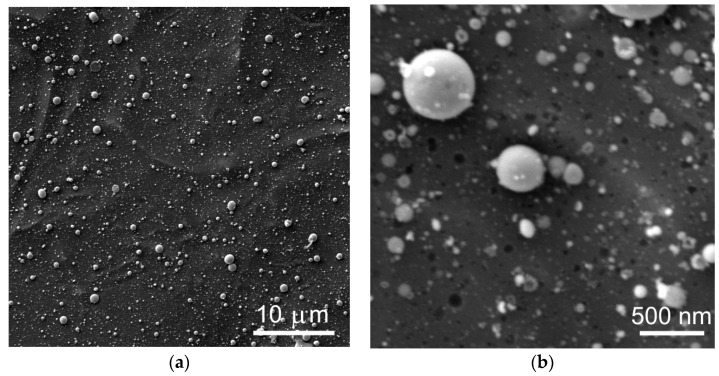
SEM images with (**a**) low and (**b**) higher magnification for the surface of an n^+^p-Si wafer covered with a 20 nm-thick catalytic a-MoS*_x_*/NP-Mo film.

Figure 8a shows SEM images for the surface of the a-MoS_x_/NP-Mo//n+p-Si sample containing a relatively thick a-MoS*_x_*/NP-Mo film. When forming a MoS_x_/NP-Mo film of a thickness of 20 nm, the surface of the sample accumulated rounded micron, submicron, and nanometric particles. The particles were retained on the surface due to sufficiently good adhesion. Micron and submicron particles were formed as a result of explosive boiling and splashing of the liquid layer during the laser ablation of the MoS_2_ target. In Figure 8b, nano-sized rounded regions with dark contrast probably originated at locations where the film surface was bombarded by high-speed particles from the laser plume. Submicron and nanometric particles with very high velocities could form craters on the film surface and be reflected or rebound from the surface. At the same time, the continuity of the a-MoS*_x_*/NP-Mo film on the silicon surface was maintained, which was confirmed by the results of a cross-section TEM study for the a-MoS*_x_*/NP-Mo//n^+^p-Si sample.

**Figure 9:**
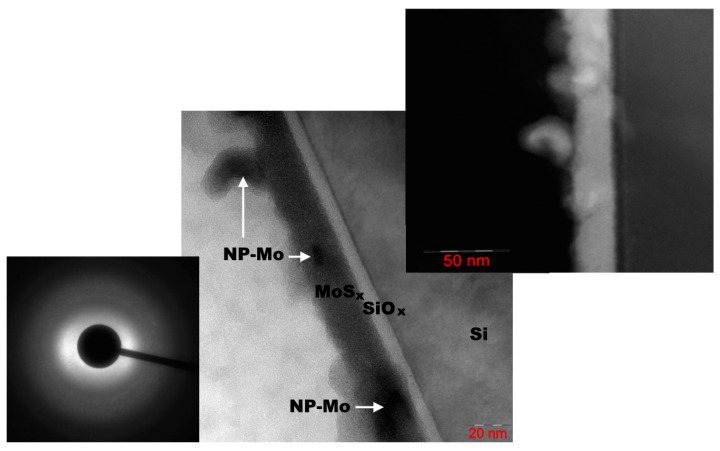
TEM image of a 20 nm thick a-MoS*_x_*/NP-Mo film prepared by PLD on n^+^p-Si. The inserts show the SEAD pattern (left) and the HAADF image (right) of the film.

Figure 9 shows a SEAD pattern and TEM and HAADF images obtained for the a-MoS*_x_*/NP-Mo//n^+^p-Si sample (with a 20 nm thick film) after air exposure of the sample for several days following PLD. It can be seen that the a-MoS*_x_*/NP-Mo film consists of a relatively dense packing of rounded nanoparticles with an amorphous structure and of a size of about 20 nm. As a result, the film had surface irregularities of up to 15 nm in height. The Mo nanoparticles were 10−20 nm in size; they were embedded in the volume of the film or located on its surface. The nanoparticles were coated with a thin layer of a-MoS*_x_*. SAED analysis revealed the amorphous structure of the a-MoS*_x_* matrix. EDX measurements indicated that the ratio S/Mo of atomic concentrations in different areas of the film varied between 1.84 and 2.3. The difference between the XPS and EDS results could be because EDS detected both molybdenum in the a-MoS*_x_* matrix and the Mo metal nanoparticles. Oxygen concentration in different areas of the a-MoS*_x_*/NP-Mo film varied from 5 to 15 at.%. Figures S3 and S4 present the depth profile of the element distribution and mapping for this sample.

**Figure 10:**
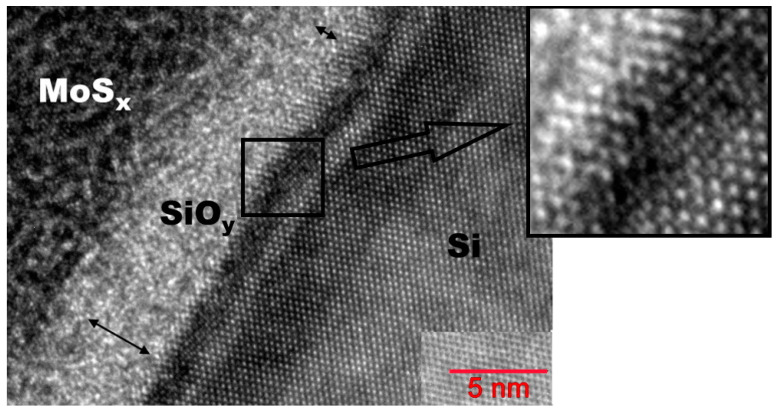
HRTEM image of an interface layer of a-MoS*_x_*/NP-Mo film with Si substrate subjected to pulsed laser P doping. The insert shows the nanocrystalline character of the laser-treated Si surface.

A thin silicon oxide layer (2−4 nm thickness) was formed between the a-MoS*_x_*/NP-Mo film and the silicon substrate (Figure 10). In the near-surface layer, the silicon substrate had a fairly perfect crystal lattice, much in line with the RBS studies. This result pointed to the epitaxial nature of the crystallization of silicon after pulse melting under laser exposure. Yet, after solidification, the surface of the silicon had noticeable roughness and defects (nanocrystallinity), which could account for the improved adhesion of the a-MoS*_x_*/NP-Mo film to the silicon wafer subjected to pulsed laser treatment.

### 3.3. Performance of a-MoS_x_/NP-Mo//n^+^p-Si Photocathodes in PEC HER

**Figure 11:**
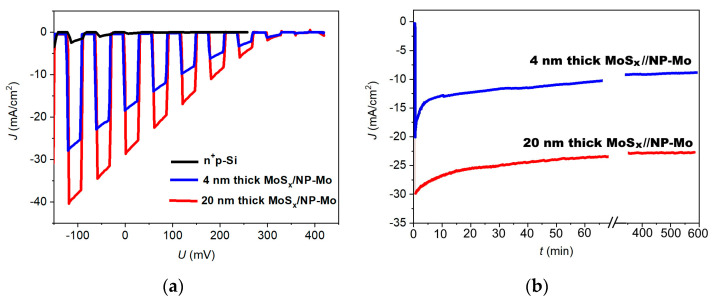
(**a**) LSV curves for n^+^p-Si without and with thin and thicker a-MoS*_x_*/NP-Mo catalytic films; (**b**) Chronoamperometric curves for n^+^p-Si covered with thin and thicker a-MoS*_x_*/NP-Mo catalytic films. The curves were measured under (**a**) chopped and (**b**) continuous illumination with an intensity of 100 mW/cm^2^.

Figure 11a presents linear voltammograms for three different photoelectrodes measured in an H_2_SO_4_ solution with the light flux from a Xe lamp turned on and off. The n^+^p-Si electrode had weak efficiency in the photo-stimulated HER. Hydrogen was released only at negative potential values. As a relatively thin a-MoS*_x_*/NP-Mo film was applied to an n^+^p-Si electrode, a significant increase in the photo-stimulated HER activity was observed. Hydrogen evolution began at a positive potential of 390 mV; the photocurrent density at zero potential reached 18.2 mA/cm^2^. Increasing the thickness of the catalyst film from 4 to 20 nm caused a further growth in the photocurrent density at zero potential, which reached 28.7 mA/cm^2^.

The temporal stability of the photocathodes was investigated under constant illumination. Figure 11b shows the results of photocurrent measurements at zero potential. It can be seen that, for a 4 nm thick a-MoS*_x_*/NP-Mo film, the photocurrent density decreased approximately 1.5-fold after 10 min. Then, the photocurrent density declined more slowly: after 600 min, the photocurrent density was 10 mA/cm^2^. In the case of a thicker film, the photocurrent density did not decrease as quickly as for the thinner film. A noticeable drop in the photocurrent density lasted for about 20 min; the current almost stabilized after about 60 min at ~25 mA/cm^2^. This value was maintained for more than 10 h.

**Figure 12:**
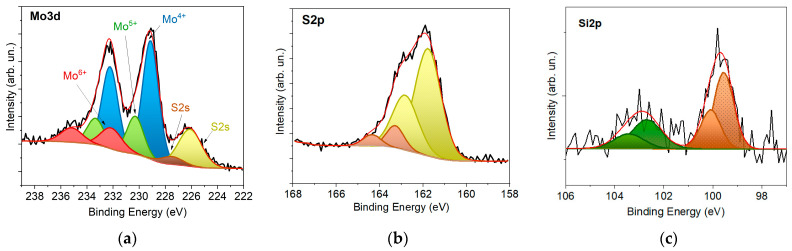
XPS (**a**) Mo3d, (**b**) S2p, and (**c**) Si2p spectra for the surface of a-MoS*_x_*/NP-Mo//n^+^p-Si cathode (with a 4 nm thick film), measured after testing in a photoelectrochemical cell for 20 min.

Figure 12 shows the results of an XPS study of the n^+^p-Si photocathode surface with a thin a-MoS*_x_*/NP-Mo film after its application in PEC HER for 20 min. Comparing the data with those in Figure 5, one can see noticeable changes in the chemical state of both the a-MoS*_x_*/NP-Mo film and the silicon wafer. The most dramatic changes occur in the state of the sulfur atoms. In the S2p spectrum, the relative intensity of the low-energy doublet corresponding to the terminal states of sulfur in MoS_x≥3_ compounds increased significantly. This doublet could also correspond to the states of sulfur in the MoS_x~2_ amorphous matrix. The change in the chemical state of sulfur was accompanied by the removal of some sulfur from the a-MoS*_x_*/NP-Mo film. As a result, the film composition was described by the formula MoS_1.8_. Several studies have observed modifications in the chemical state of sulfur on the surface of electrocatalytic MoS_x≥3_ films [24,25]. These changes are likely to occur quite rapidly after the beginning of the electrochemical investigations in the thin surface layer of an amorphous MoS*_x_* film; they are not expected to cause the degradation of catalytic properties as the time increases.

Alongside the modification of the chemical state of sulfur, film oxidation occurred with the formation of Mo^5+^ and Mo^6+^ states. The surface of the Mo nanoparticles was also subjected to oxidation, which caused the almost complete disappearance of the Mo^0^ peaks. Molybdenum oxides could form in both an acid solution during HER and after exposing the chemically active surface of a-MoS*_x_* film to air and washing it in water following an electrochemical cell examination. The formation of molybdenum oxides on the a-MoS*_x_* film surface during PEC HER could reduce the surface concentration of active sites forming on sulfur atoms/dimers. During the formation of molybdenum oxides in the bulk of a-MoS_x_ film, the resistance to electric current flow could increase in the latter. Another factor potentially impeding the performance of a-MoS*_x_*/np-Mo//n^+^p-Si photocathode is silicon oxidation. The XPS Si2p spectrum shows an increase in the intensity of the peak around 103 eV. Increasing the thickness of the SiO*_y_* layer can raise the resistance to the electric current through the interface.

**Figure 13:**
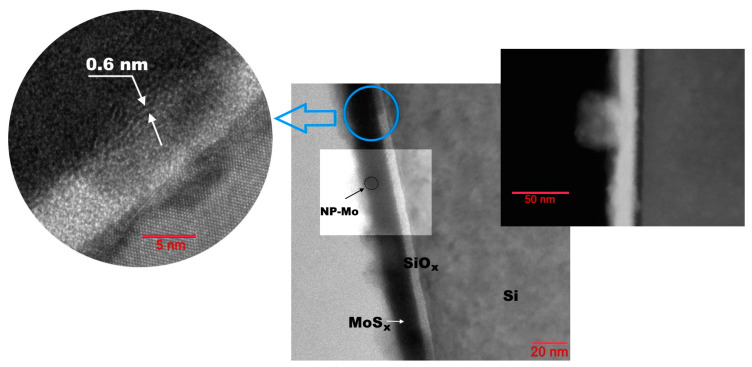
Cross section HRTEM (left), TEM (center), and HAADF (right) images for a-MoS*_x_*/NP-Mo//n^+^p-Si photocathode tested in a photoelectrochemical cell for 20 min.

Figure 13 presents the results of studying the surface layer structure of a-MoS*_x_*/NP-Mo//n^+^p-Si photocathode with a thicker a-MoS*_x_*/NP-Mo film after testing in the electrochemical cell. The cathode was tested for 20 min at zero potential under Xe lamp illumination. Figures S5 and S6 show the surface layer depth distribution of the elements and their mapping. The investigation revealed no significant changes in the a-MoS*_x_*/NP-Mo film thickness and structure. An EDS study of the film composition showed that the surface layer of the a-MoS*_x_*/NP-Mo film could have a reduced concentration of sulfur with the formation of the MoS_1.6_ compound, with the oxygen concentration increasing to 30 at.%. When the analyzing X-ray beam was shifted into the depth of the film, the film composition was described by the formula MoS_2.5_–MoS_2.8,_ and the oxygen concentration decreased to 14 at.%.

The silicon oxide layer thickness after PEC HER testing increased by no more than 1 nm. The depth distribution of sulfur no longer showed a spike in the concentration at the SiO*_y_*/Si interface (Figure S5). Therefore, both oxygen and sulfur atoms could have migrated at the SiO*_y_*/Si interface during PEC HER. Notably, crystallization of the a-MoS*_x_* matrix could occur near the a-MoS*_x_* interface with SiO*_y_*_._ This process leads to the formation of a laminar packing of atoms characteristic of the MoS_2_ turbostratic phase. The distance between the atomic planes was ~0.6 nm; they were oriented parallel to the silicon surface (Figure 13). This effect could be due to specific processes in PEC and/or the effect of the electron beam [55]. If crystallization occurred during the PEC process, for example, due to stress relaxation in a metastable a-MoS*_x_* matrix, the MoS_2_ crystalline film could produce a protective effect preventing intensive corrosion of the silicon electrode [55,56].

## 4. Discussion

The findings suggest that the pulsed laser doping of p-Si wafers in an orthophosphoric acid solution involved the melting of silicon and the introduction of phosphorus atoms formed through acid decomposition during laser heating near the silicon surface. Phosphorus penetration proceeded by the mechanism of diffusion in liquid silicon. After melting, epitaxial growth of the silicon crystal structure occurred. The instability of the melting front caused the formation of nanometric irregularities, which contained lattice defects, on the silicon surface. Probably, the adhesion of the f-MoS*_x_*/NP-Mo film to such a silicon surface may be stronger than to the atomically smooth surface of the virgin p-Si single crystal.

We had no opportunity to measure the phosphorus concentration in the silicon after laser doping. Yet the electrical measurements indicated the formation of a structure exhibiting a rectification effect and increasing current when the laser-doped silicon wafer surface was illuminated. Therefore, a sufficiently large number of phosphorus atoms could have been introduced into p-Si during the laser doping. The conductivity type in the surface layer of silicon changed from hole to electronic, and an n^+^p junction formed at some depth. The literature shows that the penetration depth of P atoms reaches one micrometer [45].

**Figure 14:**
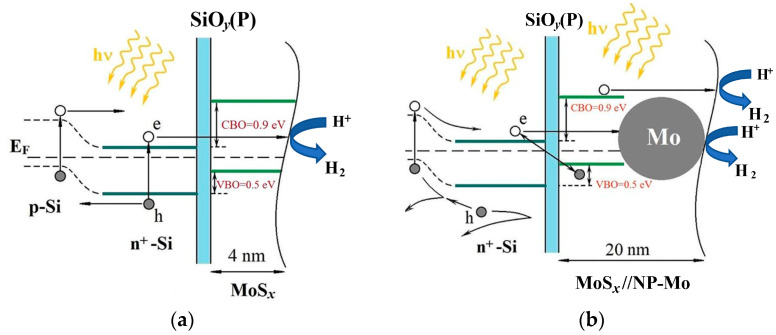
Energy band alignment and PEC HER mechanism for a-MoS*_x_*/NP-Mo//n^+^p-Si photocathodes covered with (**a**) 4 nm thick and (**b**) 20 nm thick a-MoS*_x_*/NP-Mo films. Explanations are given in the text.

Figure S7 shows the electron energy spectra for the valence band of the a-MoS*_x_*/NP-Mo and P-doped p-Si. It can be seen that the position of the Fermi level in the samples indicates a possibility of an energy barrier at the a-MoS*_x_*/NP-Mo and n^+^-Si interface. Band-off-set studies for an a-MoS*_x_*/NP-Mo thin film prepared by PLD on n^+^p-Si confirmed this fact and showed that the *VBO* was 0.5 eV. Figure 14a presents a scheme of the mutual arrangement of energy bands that can be formed in a-MoS*_x_*/NP-Mo//n^+^p-Si photocathodes. The band gap width of the silicon was set at 1.1 eV. For the energy band width of the a-MoS*_x_*/NP-Mo film, a value was chosen that is typical for amorphous a-MoS*_x_*, ~1.5 eV [5]. When illuminating the photocathode with a ~4 nm thick catalyst film, tunneling of electrons, which accumulate in n^+^-Si layer, is possible to the catalytically active surface of a-MoS*_x_*/NP-Mo. In such a case, the efficiency of PEC HER will be determined by the generation, recombination, and transfer of electrons and holes formed in the n^+^-p junction region under the light flux. Increasing the thickness of the SiO*_y_* layer, which can occur during PEC HER will diminish the efficiency of electron tunneling to the a-MoS*_x_*/NP-Mo surface, and thus worsen the characteristics of the photocathode in hydrogen production.

If the thickness of a-MoS*_x_*/NP-Mo film is increased to 20 nm, a different PEC HER mechanism should be expected since electron tunneling from silicon through such a film is unlikely. The most probable mechanism of a-MoS*_x_*/NP-Mo//n^+^p-Si photocathode functioning with a 20 nm thick catalyst film should be suggested as a Z scheme (Figure 14b). According to this scheme, the photo-stimulated HER process proceeds with the participation of electrons formed in the a-MoS*_x_*/NP-Mo film during illumination. The electrons formed in n^+^p-Si probably participate in the recombination process with the holes formed in the a-MoS*_x_*/NP-Mo film. This process occurs at the a-MoS*_x_*/NP-Mo interface with silicon, and the P-doped SiO*_y_*(P) layer may influence this process to some extent.

The XPS investigation of the a-MoS*_x_*/NP-Mo//n^+^p-Si sample with a thin a-MoS*_x_*/NP-Mo (4 nm thick) film showed (Figure 5) that the formed silicon oxide layer is inhomogeneous and can probably contain silicon inclusions heavily doped with phosphorous. Such a SiO*_y_*(P)/Si(P) layer can ensure electron migration through the oxide layer to the SiO*_y_*/a-MoS*_x_* interface while creating a barrier to holes. Thus, the holes formed in n^+^-Si upon illumination will not be able to move into an a-MoS*_x_*/NP-Mo film but rather migrate to p-Si. This assumption is supported by investigating the impedance of an a-MoS*_x_*/NP-Mo//n^+^p-Si photocathode. The study showed that the heterojunction between an a-MoS_x_/NP-Mo film and n^+^-Si has a sufficiently low resistance (0.45 Ω) for charge transfer (Figure S8).

Previously, it was found that Mo nanoparticles can increase the HER efficiency of a-MoS*_x_* due to a synergistic effect [32]. The thin a-MoS*_x_*/NP-Mo catalytic film contained a relatively low surface concentration of Mo nanoparticles. Therefore, their influence on the photo-HER efficiency may not be pronounced. Obviously, the Z-mechanism of photo-activated HER also occurs in the a-MoS*_x_*/NP-Mo//n^+^p-Si cathode possessing 4 nm thick catalytic film. However, we assumed that its contribution to photo-HER efficiency is less significant than the contribution from electron tunnelling through the energetic barrier. For Z-scheme, the electron flux to the a-MoS*_x_* catalyst surface depends on the thickness of the photo-active/semiconductor catalyst. The thin a-MoS*_x_* catalytic film had a thickness of no more than 4 nm, but the photocurrent was only ~1.5 times less than the photocurrent measured for the a-MoS*_x_*/NP-Mo catalytic film about 20 nm thick. We assumed that for a thin a-MoS*_x_* film the effect of electron tunnelling from photo-active n^+^p-Si to a-MoS_x_ surface layer could manifest itself. It is not yet clear how Mo nanoparticles affect the current flow in an a-MoS*_x_*/NP-Mo//n^+^p-Si photocathode. Possible photogenerated electron tunnelling from silicon to a Mo nanoparticle through thin layers of an a-MoS*_x_* matrix to the surface of the a-MoS*_x_* catalyst for participation in HER cannot be ruled out. Further work is required here.

Comparative study with a Pt//n^+^p-Si photocathode was carried out for a reliable assessment of the efficiency of the a-MoS_x_/NP-Mo catalyst application. The method for preparing a control Pt//n^+^p-Si cathode is described in the supplementary materials. The on-set potential for a Pt//n^+^p-Si photocathode was 350 mV, with the current density not exceeding 16 mA/cm^2^ at zero potential (Figure S9). This points to the high performance of the obtained a-MoS*_x_*/NP-Mo//n^+^p-Si photocathodes. For the best a-MoS_x_/NP-Mo//n^+^p-Si photocathode, the on-set potential was 390 mV, with the current density reaching 28.7 mA/cm^2^ at zero potential. The photocurrent density stabilized at 25 mA/cm^2^ (at zero potential) to remain at this level for 10 h. The a-MoS*_x_*/NP-Mo//n^+^p-Si photocathodes were created using physical laser techniques of p-Si surface treatment and the deposition of a catalyst made of the natural MoS_2_ material. These physical techniques give ample opportunities for further modifying the obtained materials and optimizing the formation modes of such photocathodes.

When using (photo)(electro)chemical techniques for obtaining and depositing amorphous a-MoS*_x_* or crystalline MoS_2_ films on Si wafers with an n-p junction, photocathodes were created whose properties were either worse or slightly better than those of the electrodes described in this work. For example, Laursen et al. [44] and Benck et al. [3] created an MoS*_x_*/MoS_2_/Mo//n^+^p-Si photocathode using a combined technique. At first, a p-Si wafer was doped at a high temperature (900 °C) with POCl3 as the P-source. The thin-film Mo precursor for MoS_2_ was then sulfurized, followed by the formation and photo-activated electrodeposition of MoS*_x_* in an (NH_4_)_2_MoS_4_ solution. The on-set potential of the MoS*_x_*/MoS_2_/Mo/n^+^p-Si was 0.334 V (RHE), and the photocurrent density at 0 V was 12–15 mA/cm^2^. Fan et al. [9] fabricated highly efficient (0.4 V vs. RHE on-set potential and 35.6 mA/cm^2^ saturated photocurrent measured under 100 mA/cm^2^ Xe lamp illumination) and stable photocathodes by integrating crystalline MoS_2_ catalyst with ~2 nm Al_2_O_3_ protected n^+^p-Si. The sputtering technique, using a MoS_2_ target and high-temperature post-annealing (800 °C), led to a vertically standing, conformal, and crystalline nano-MoS_2_ layer on an Al_2_O_3_/n^+^p-Si photocathode. Surface pyramid texture and P-doping were realized by chemical treatment of p-Si wafer. Zhang S. et al. [37] designed a green and mild route to insert an N-doped carbon (CN) layer between Si and MoS*_x_*, which improves the transfer of interfacial carriers and avoids unnecessary absorption of incident light. The a-MoS*_x_*/CN//pn-Si hybrid photocathode delivered excellent performance (0.23 V on-set potential and 10 mA/cm^2^ photocurrent at 0 V (RHE) in 0.5 M H_2_SO_4_ under the condition of simulated sunlight). The fabricated MoS*_x_* catalytic films had n-type conductivity.

The laser-doped Si wafer surface could not have the same optical properties (low reflection coefficient) as the pyramid-textured surface specifically created by chemical etching. The textured surface problem can be solved in the long term by using other laser treatment modes, for example, by employing lasers with shorter pulse durations [57]. The chosen mode of pulsed laser doping may not be optimal for obtaining a high-quality n^+^p-junction. Moreover, the photovoltaic characteristics, particularly the fill factor, can probably be improved by post-treatment of the laser-doped samples, for instance, thermal or pulse lamp annealing. It is also necessary to understand more deeply the issue of the influence of silicon oxide, which is formed at the PLD stage and, possibly, in the electrolyte, on the perfection of the a-MoS*_x_*/NP-Mo//n^+^p-Si photocathode.

## 5. Conclusions

The a-MoS_x_/NP-Mo thin films obtained the highly nonequilibrium conditions of pulsed laser deposition from a MoS_2_ target have the qualitative electrocatalytic properties for HER activation. These properties are due to the high concentration of active sites in the MoS*_x_* amorphous matrix and the synergistic effect of Mo nanoparticles on the efficiency of HER occurring on the active sites. These films can be successfully used, despite p-type conductivity, to form silicon-based p-n junction photocathodes for hydrogen production in PEC HER in an acid solution. Pulsed laser doping with phosphorus atoms was effectively applied in obtaining an n^+^p-junction in the near-surface region of silicon to simplify the technology of producing an n^+^p-junction in p-Si. The most impressive performance of an a-MoS*_x_*/NP-Mo//n^+^p-Si photocathode was achieved using a-MoS*_x_*/NP-Mo films with ~20 nm thickness. The on-set potential was 390 mV, the current density at zero potential reaching 28.7 mA/cm^2^. On such a photocathode, the photo-stimulated HER probably proceeded according to the Z-scheme under the specific influence of the SiO*_y_*(P) interlayer. This nanolayer formed at the interface between a-MoS*_x_*/NP-Mo and n^+^-Si at the stage of catalytic film deposition and changed weakly during PEC HER. In this work, the developed a-MoS*_x_*/NP-Mo//n^+^p-Si photocathodes and approbated laser-based technique for Si surface modification (P doping) and a-MoS*_x_*/NP-Mo catalytic film deposition are considered as a potential green pathway toward high-effective hydrogen production by solar energy conversion.

## Data Availability

Not applicable.

## Supplementary Materials

The following supporting information can be downloaded at: https://www.mdpi.com/article/10.3390/nano12122080/s1, Figure S1: ERD spectra for virgin p-Si (blue), P-doped p-Si (red), P-doped p-Si after 10% HF treatment (green), and pure silicon (black). No clear differences were found in these spectra. For all the samples, the content of H atoms on the surface was ~2.5 × 10^15^ cm^−2^; Figure S2: Electric current vs. voltage dependences measured for the virgin p-Si and P-doped p-Si. For the P-doped p-Si, the *I(U)* curve was changed when the sample was illuminated with a light of optical microscope lamp that indicates the existence of n^+^p-junction; Figure S3: Cross-section electron image and depth distribution (bottom) of Si, O, S, and Mo in the surface layer of a-MoS*_x_*/NP-Mo//n^+^p-Si sample. A peak is seen in the depth distribution of sulfur at a depth exceeding the thickness of the silicon oxide layer. The growth of the Si-O layer proceeded due to migration of silicon atoms through a thin layer Si-O-S layer, which could be formed after the adsorption of oxygen and sulfur during PLD; Figure S4: Maps of Si, O, Mo, and S distributions in the surface layer of a-MoS*_x_*/NP-Mo//n^+^p-Si sample. It should be noted that in the electron images the MoS*_x_* film matrix consists of clusters with laminar packing of atomic planes. The interplanar distance is ~0.6 nm. The cluster sizes are 5–10 nm. The crystallization of the amorphous MoSx film was due to long-term electron beam irradiation of the sample during TEM study; Figure S5: Cross-section electron image and depth distribution (bottom) of Si, O, S, and Mo in the surface layer of a-MoS*_x_*/NP-Mo//n^+^p-Si sample. The measurements were carried out after photoelectrochemical testing of the photocathode for 20 min; Figure S6: Maps of Si, O, Mo, and S distributions in the surface layer of a-MoS*_x_*/NP-Mo//n^+^p-Si sample after photoelectrochemical testing of the photocathode for 20 min; Figure S7: XPS valence band spectra for (a) 20 nm thick a-MoS*_x_* film and (b) P-doped p-Si wafer; Figure S8: Experimental and fitted EIS curves for a-MoS*_x_*/NP-Mo//n^+^p-Si photocathode. Equivalent circuit includes ohmic resistance for electric current throughout the substrate (R2) and series-connected resistance for current through the n+-p junction (R1), a-MoS*_x_*/NP-Mo–Si interface (R3), and a-MoS*_x_*/NP-Mo contact area/interfaces with electrolyte (R4). Resistances were measured in Ohm units. The equivalent circuit contains constant phase elements (CPEs) which were added in the frame of the conventional approach to modeling photocathode with heterojunction structure; Figure S9: LSV curve measured under light chopping for n^+^p-Si photocathode covered with Pt film.
