# Supplementary material for: Pulsed Laser Phosphorus Doping and Nanocomposite Catalysts Deposition in Forming a-MoSx/NP-Mo//n+p-Si Photocathodes for Efficient Solar Hydrogen Production
Source: Nanomaterials (Basel). 2022 Jun 16;12(12):2080. doi: 10.3390/nano12122080 (PMC9227624; doi:10.3390/nano12122080)
Supplement: Supplementary file 1 [file nanomaterials-12-02080-s001.zip › nanomaterials-1754092-supplementary.pdf]

Supplementary Materials

Pulsed Laser Phosphorus Doping and Nanocomposite Catalysts Deposition in Forming a-MoS_x/NP-Mo//n^+p-Si Photocathodes for Efficient Solar Hydrogen Production

Vyacheslav Fominski [1,*], Maxim Demin [2], Dmitry Fominski [1], Roman Romanov [1], Oxana Rubinkovskaya [1], Petr Shvets [2] and Aleksandr Goikhman [2]

[1] National Research Nuclear University MEPhI (Moscow Engineering Physics Institute), Kashirskoe sh. 31, 115409 Moscow, Russia; dmitryfominski@gmail.com (D.F.); limpo2003@mail.ru (R.R.); oxygenofunt@gmail.com (O.R.)
[2] Immanuel Kant Baltic Federal University, A. Nevskogo St. 14, 236016 Kaliningrad, Russia; sterlad@mail.ru (M.D.); pshvets@kantiana.ru (P.S.); aygoikhman@gmail.com (A.G.)
* Correspondence: vyfominskij@mephi.ru

Features of pulsed laser doping of p-Si wafer with phosphorus

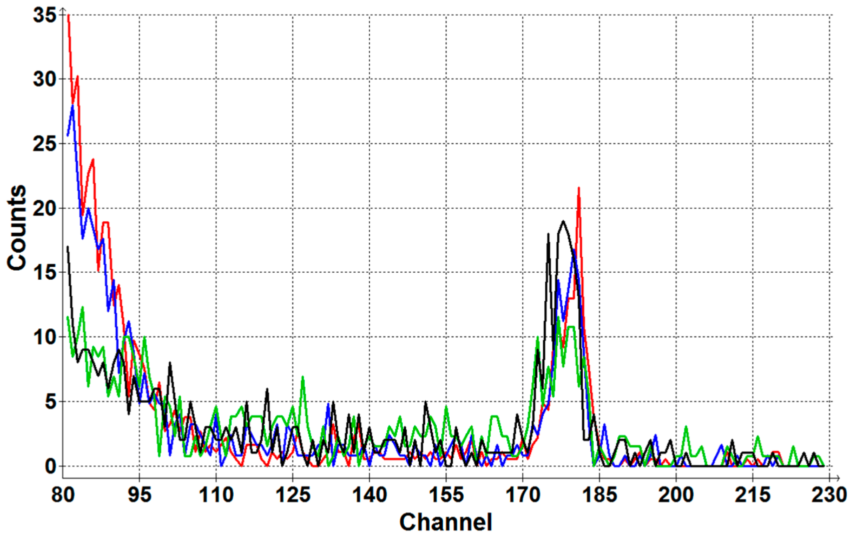

Figure S1. ERD spectra for virgin p-Si (blue), P-doped p-Si (red), P-doped p-Si after 10% HF treatment (green), and pure silicon (black). No clear differences were found in these spectra. For all the samples, the content of H atoms on the surface was ~2.5×10^{15} cm^{-2}.

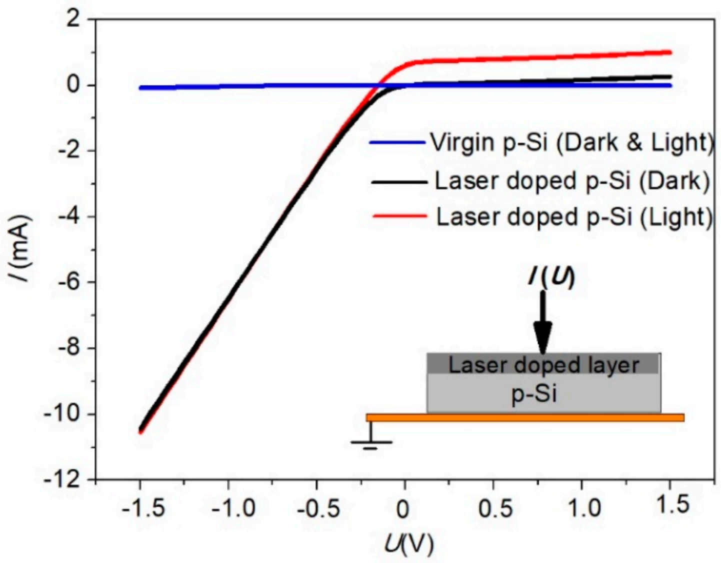

Figure S2. Electric current vs. voltage dependences measured for the virgin p-Si and P-doped p-Si. For the P-doped p-Si, the I(U) curve was changed when the sample was illuminated with a light of optical microscope lamp that indicates the existence of n^+p-junction.

Depth distribution of elements in a-MoS_x/NP-Mo//n[+]p-Si photocathode after pulsed laser deposition of catalytic film

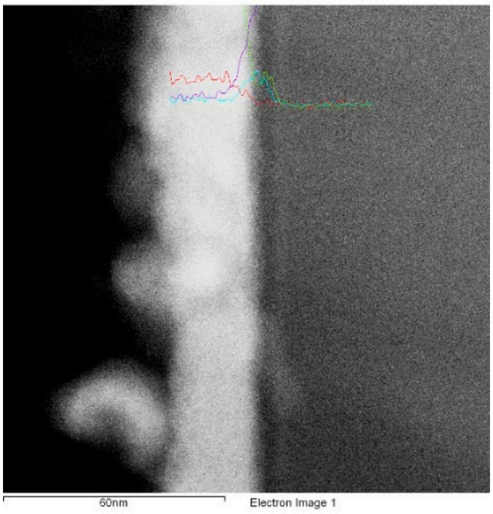

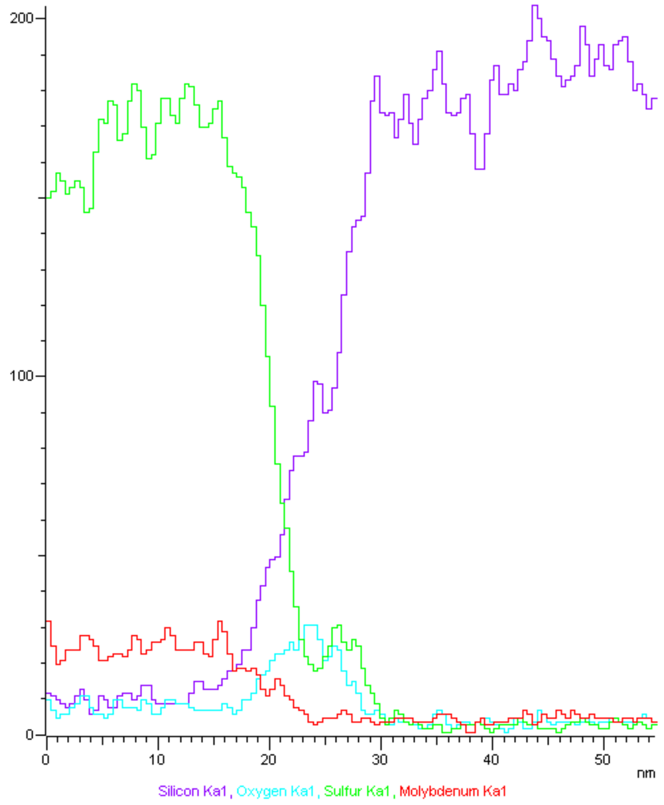

Figure S3. Cross-section electron image and depth distribution (bottom) of Si, O, S, and Mo in the surface layer of a-MoS_x/NP-Mo//n[+]p-Si sample. A peak is seen in the depth distribution of sulfur at a depth exceeding the thickness of silicon oxide layer. The growth of the Si-O layer proceeded due to migration of silicon atoms through a thin layer Si-O-S layer which could be formed after the adsorption of oxygen and sulfur during PLD.

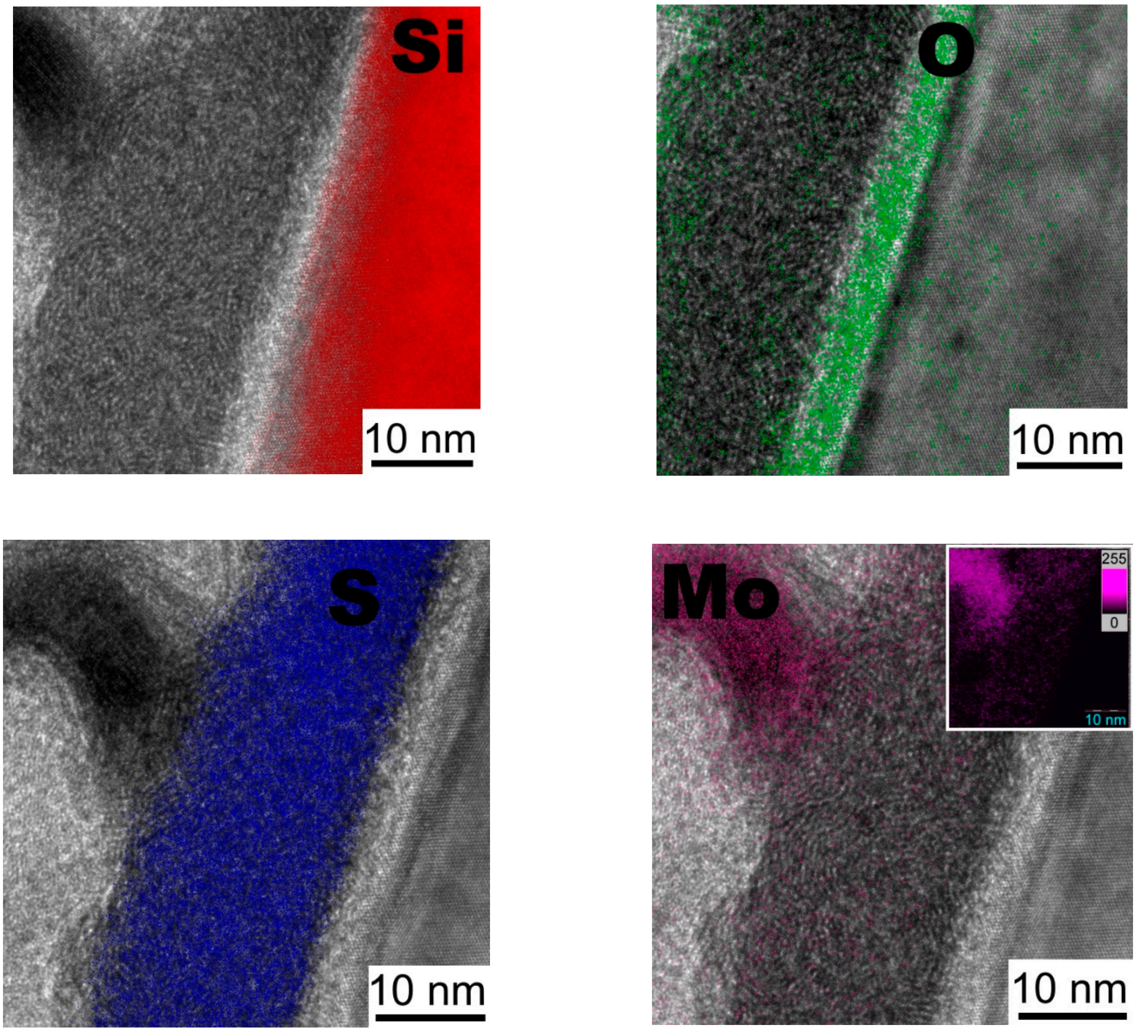

Figure S4. Maps of Si, O, Mo, and S distributions in the surface layer of a-MoS_x/NP-Mo//n^+p-Si sample. It should be noted that in the electron images the MoS_x film matrix consists of clusters with laminar packing of atomic planes. The interplanar distance is ~0.6 nm. The cluster sizes are 5 – 10 nm. Crystallization of the amorphous MoSx film was due to long-term electron beam irradiation of the sample during TEM study.

Depth distribution of elements in a-MoS_x/NP-Mo//n^+p-Si photocathode after photoelectrochemical testing

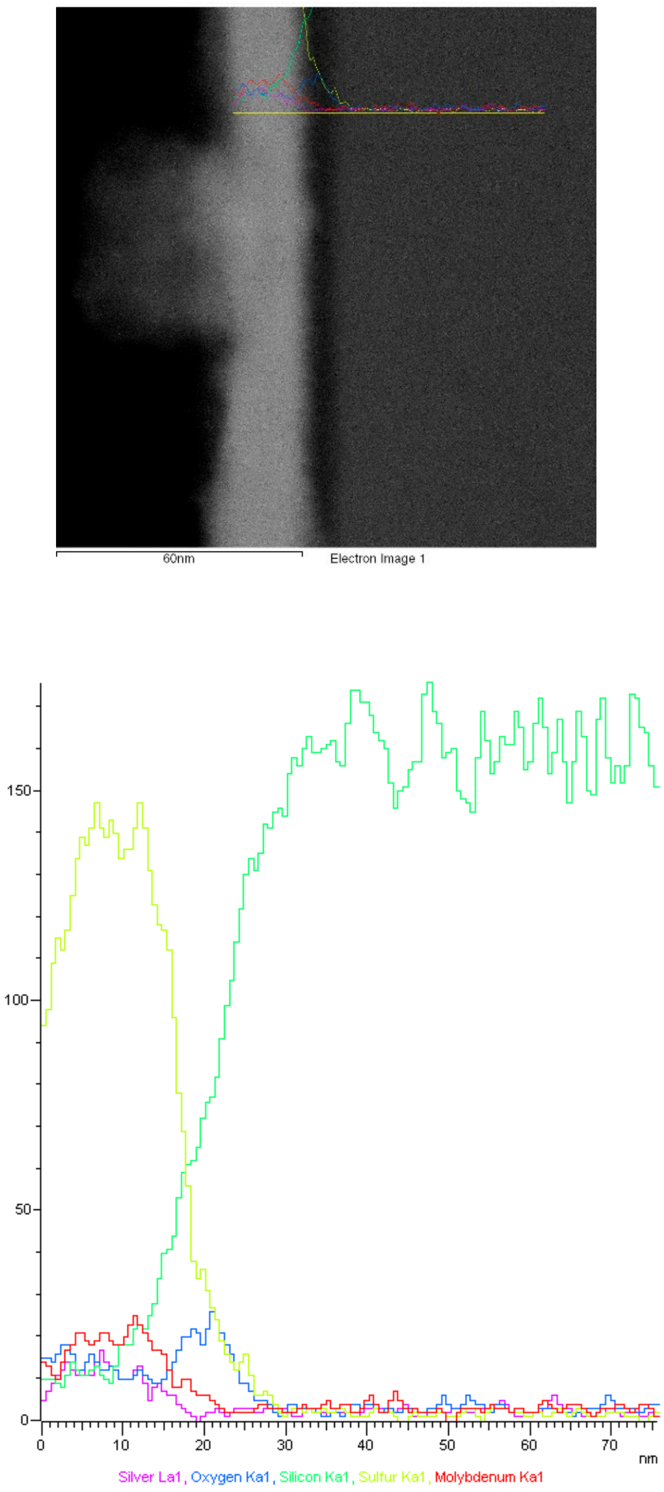

Figure S5. Cross-section electron image and depth distribution (bottom) of Si, O, S, and Mo in the surface layer of a-MoS_x/NP-Mo//n^+p-Si sample. The measurements were carried out after photoelectrochemical testing of the photocathode for 20 min. (Ag distribution should not be considered).

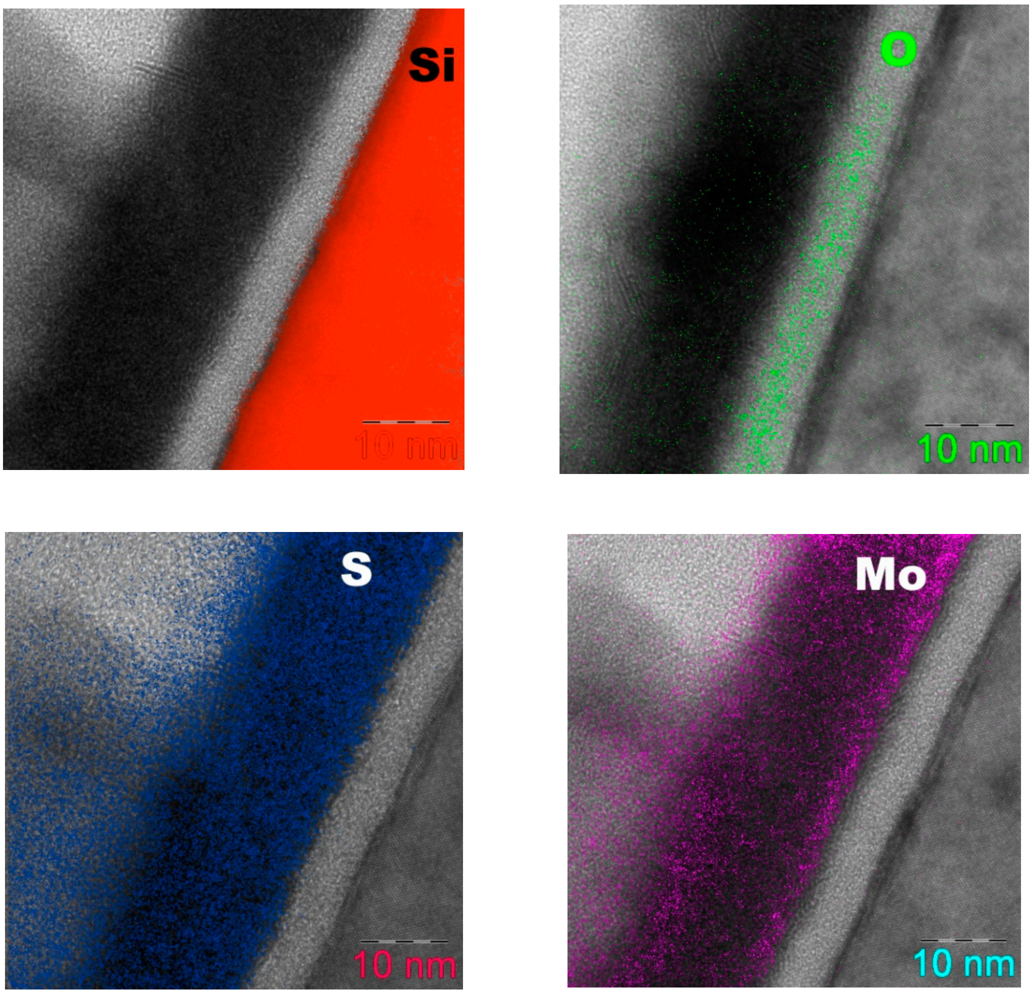

Figure S6. Maps of Si, O, Mo, and S distributions in the surface layer of a-MoS_x/NP-Mo//n^+p-Si sample after photoelectrochemical testing of the photocathode for 20 min.

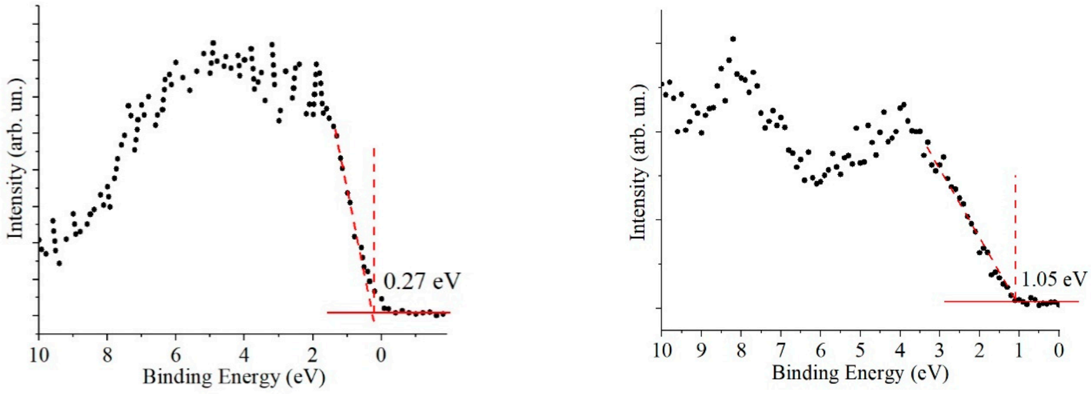

(**a**) (**b**)

Figure S7. XPS valience band spectra for (**a**) 20 nm thick a-MoS_x film and (**b**) P-doped p-Si wafer.

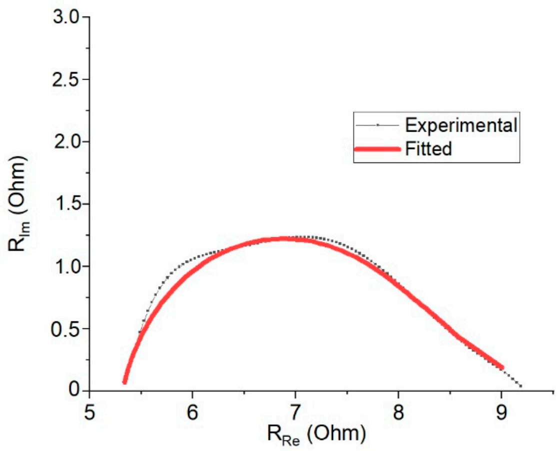

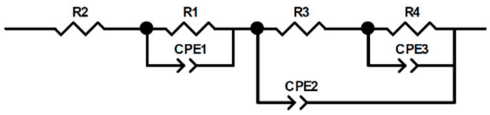

**Element**	**Value**
**R2**	**5.32**
**R1**	**3**
CPE1-T	0.00025
CPE1-P	0.85
**R3**	**0.45**
**R4**	**0.3**
CPE3-T	0.05
CPE3-P	0.99
CPE2-T	0.0055
CPE2-P	0.995

Figure S8. Experimental and fitted EIS curves for a-MoS_x/NP-Mo//n^+p-Si photocathode. Equivalent circuit includs ohmic resistance for electric current throughout the substrate (R2) and series-connected resistance for current through the n^+-p junction (R1), a-MoS_x/NP-Mo—Si interface (R3), and a-MoS_x/NP-Mo contact area/interfaces with electrolyte (R4). The resistance was measured in Ohm unit. The equivalent circuit contains constant phase elements (CPEs) which were added in the frame of the conventional approach to modeling photocathode with heterojunction structure.

Preparation of Pt/n⁺p-Si photocathode and its PEC HER performance

The p-Si wafer was preliminarily subjected to pulsed laser doping with phosphorus in orthophosphoric acid. The same regimes of pulsed laser irradiation were used as in the preparation of a n^+p-junction for a-MoS_x/NP-Mo//n^+p-Si photocathode. Instead of a-MoS_x//NP-Mo film, a platinum film ~50 nm thick was deposited on the surface of n^+p-Si previously subjected to chemical treatment in buffered HF. For Pt deposition, the pulsed laser deposition under vacuum conditions was used. Pt plate was used as a target for pulsed laser ablation. Laser radiation had a wavelength of 1064 nm, a pulse duration of 15 ns, a pulse repetition rate of 20 Hz, a pulse energy of ~ 90 mJ, and a laser fluence of ~ 10 J/cm^2. The PLD vacuum chamber was pumped out by a turbomolecular pump to a pressure of ~10^{-3} Pa. The back of the n^+p-Si samples was covered by a thin Au layer to obtain an Ohmic contact with p-Si. A 30 nm-thick Au film was prepared by PLD from the Au target at room temperature of the samples. To investigate the PEC characteristics of Pt//n^+p-Si cathode in HER, it was illuminated by radiation from Xe lamps with a power of 100 W in a 0.5 M H_2SO_4 aqueous solution. The light intensity was maintained at 100 mW/cm^2. LSV curve for Pt//n^+p-Si cahode is shown in Fig. S9. The on-set potential for a Pt//n^+p-Si photocathode was 350 mV, with the current density not exceeding 16 mA/cm^2 at U=0 V (RHE). The use of Pt film catalyst caused the appearance of noticeable dark currents at negative potentials. The dark current could be due to the high electrocatalytic activity of platinum. However this characteristic was not realized properly in the formed photocathode. The platinum film could affect the efficiency of photo-activated processes in n^+p-Si. For the selected Pt film thickness, the reflectivity of the surface increased and the intensity of the light flux which penetrated into the depth of n+p-junction could decrease.

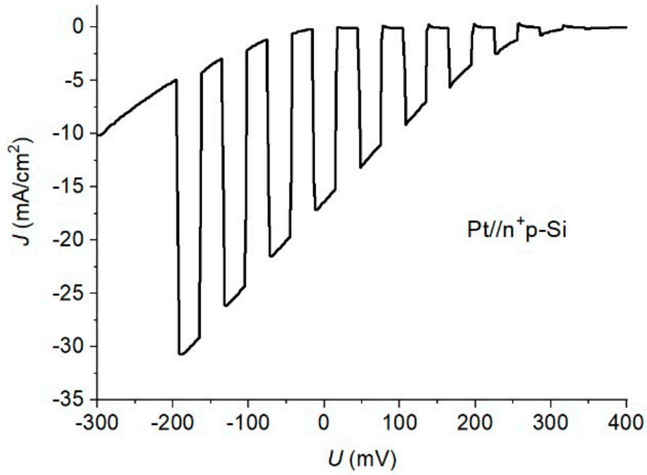

Figure S9. LSV curve measured under light chopping for n^+p-Si photocathode covered with Pt film.
